# Environmental Occurrence and Predicted Pharmacological Risk to Freshwater Fish of over 200 Neuroactive Pharmaceuticals in Widespread Use

**DOI:** 10.3390/toxics10050233

**Published:** 2022-05-03

**Authors:** John P. Sumpter, Luigi Margiotta-Casaluci

**Affiliations:** 1 Department of Life Sciences, College of Health, Medicine and Life Sciences, Brunel University London, London UB8 3PH, UK; john.sumpter@brunel.ac.uk; 2 Department of Analytical, Environmental and Forensic Sciences, School of Cancer and Pharmaceutical Sciences, King’s College London, London SE1 9NQ, UK

**Keywords:** pharmaceuticals in the environment, environmental risk assessment, behaviour, fish, ecotoxicology, mixture toxicology, predictive toxicology, pollution

## Abstract

There is a growing concern that neuroactive chemicals released into the environment can perturb wildlife behaviour. Among these chemicals, pharmaceuticals such as antidepressants and anxiolytics have been receiving increasing attention, as they are specifically prescribed to modify behavioural responses. Many laboratory studies have demonstrated that some of these compounds can affect various aspects of the behaviour of a range of aquatic organisms; however, these investigations are focused on a very small set of neuroactive pharmaceuticals, and they often consider one compound at a time. In this study, to better understand the environmental and toxicological dimension of the problem, we considered all pharmaceuticals explicitly intended to modulate the central nervous system (CNS), and we hypothesised that these compounds have higher probability of perturbing animal behaviour. Based on this hypothesis, we used the classification of pharmaceuticals provided by the British National Formulary (based on their clinical applications) and identified 210 different CNS-acting pharmaceuticals prescribed in the UK to treat a variety of CNS-related conditions, including mental health and sleep disorders, dementia, epilepsy, nausea, and pain. The analysis of existing databases revealed that 84 of these compounds were already detected in surface waters worldwide. Using a biological read-across approach based on the extrapolation of clinical data, we predicted that the concentration of 32 of these neuroactive pharmaceuticals in surface waters in England may be high enough to elicit pharmacological effects in wild fish. The ecotoxicological effects of the vast majority of these compounds are currently uncharacterised. Overall, these results highlight the importance of addressing this environmental challenge from a mixture toxicology and systems perspective. The knowledge platform developed in the present study can guide future region-specific prioritisation efforts, inform the design of mixture studies, and foster interdisciplinary efforts aimed at identifying novel approaches to predict and interpret the ecological implications of chemical-induced behaviour disruption.

## 1. Introduction

The sustainability of animal populations relies on the evolution and display of complex behavioural responses aimed at meeting the basic needs of the organism, such as finding resources—including food and water—surviving, and reproducing successfully. Human domination of the planet, especially recently, has led to profound changes to all ecosystems, which has often necessitated animals to rapidly adapt and change their behaviour in order to survive. A rapidly growing number of studies have reported the impact of human activities on wildlife behaviour in both aquatic and terrestrial ecosystems (recently reviewed by Wilson et al. (2020)) [1]. The range of behavioural effects is wide and includes the disruption of movement, foraging, risk-taking behaviour, communication, and breeding. For example, a meta-analysis of 208 studies on 167 aquatic and terrestrial species carried out by Doherty et al. (2021) [2] showed that disturbance by humans has widespread impacts on the movements of birds, mammals, reptiles, amphibians, fish, and arthropods. The mechanisms via which humans disrupt wildlife behaviour are also numerous and include the active modification of population densities (e.g., via fishing, hunting, etc.) and habitat structure, and the introduction of sensory pollution [1]. For example, the noise generated by human activities has well-established detrimental effects on wildlife [3], such as the disruption of respiratory and resting behaviour of humpback whales in response to whale-watching vessel noise emissions [4]. On the other hand, light pollution is known to affect both nesting behaviour of turtles and the subsequent risk of predation of the nests and of hatchlings [5,6]. 

Among the many anthropogenic stressors, chemical pollution is one of the greatest global threats for both humans [7] and wildlife [8]. There is a growing concern that chemicals released into the environment so far have modified the behaviour of wild organisms [9,10]. Although demonstrating the causal effects of chemicals on the behaviour of wildlife is very challenging, it is known that some of those chemicals have already elicited such effect. For example, behaviour-modifying chemicals are widely used for large-scale pest control and management (e.g., insect repellents, semiochemicals) [11]. In the last two decades, a specific class of chemicals has sparked a renewed interest in behavioural ecotoxicology. That is the class of psychoactive pharmaceuticals, such as antidepressants and anxiolytics. The use of psychoactive drugs in Western countries has been growing steadily in the last few decades [12,13]. One of the consequences of this increased consumption is that low concentrations of these pharmaceuticals can often be detected in the aquatic environment [14]. Many pharmacological targets of psychoactive drugs are also evolutionarily conserved in fish species; therefore, these drugs may cause behavioural alterations of aquatic wildlife as they do in humans [15]. As appropriate behavioural responses are critical for virtually any key aspect of individual survival and population sustainability, drug-induced behavioural alterations may lead to profound, non-linear, and perhaps unpredictable ecological effects [16]. The importance of this issue was first brought to light in the early 2000s with the detection of the antidepressant fluoxetine in American rivers [17,18]. Brooks et al. were the first scientists to raise the possibility that some anti-depressants acting as selective serotonin transport inhibitors (SSRIs) could be present in the aquatic environment at concentrations high enough to affect the behaviour of fish and other aquatic species [19,20,21]. Since that discovery, significant efforts have been allocated to characterize the environmental risk of fluoxetine. These efforts (and relative controversies) still persist 20 years later, with more than 140 studies on various aspects of fluoxetine environmental risk published up to 2021. Following the scientific and media attention on the problem, the effects of a few other psychoactive drugs on aquatic species were studied in the following years, including the antidepressant sertraline [22] and the anxiolytic oxazepam [23]. The latter work contributed to raising the profile and the degree of concern of the issue further.

Despite the undoubted challenges of both recording and then interpreting behavioural data, there are now many reports from many scientists that psychoactive drugs, particularly anti-depressants, can affect various aspects of the behaviour of a range of aquatic organisms. However, nearly all of these claims are based on the results of laboratory investigations; their extrapolation to the natural environment is much less certain. Moreover, these laboratory experiments have almost all involved exposing aquatic organisms, in particular, fish, to single psychoactive pharmaceuticals. Yet there is now a very substantial body of evidence showing that the aquatic environment is contaminated with many different neuroactive drugs (see later for details), as well as non-pharmaceutical pollutants potentially able to perturb animal behaviour. Thus, it is the potential behavioural effects of these complex mixtures of drugs that is the ecologically relevant scenario. 

In the present study, to better understand the environmental and toxicological dimension of the problem, we expanded our focus beyond antidepressants and anxiolytics. Specifically, we considered all pharmaceuticals explicitly intended to modulate the central nervous system (CNS), and we hypothesised that CNS-acting drugs have higher probability of perturbing animal behaviour. Using the UK pharmaceutical market as the case study, we generated a first comprehensive assessment of the pharmacological risk posed by neuroactive pharmaceuticals to wild fish. By defining the current eco-pharmacological landscape, our results provide an initial knowledge platform to guide future research efforts aimed at predicting and interpreting the ecological implications of chemical-induced behaviour disruption using a systems perspective.

## 2. Materials and Methods

### 2.1. Identification of Neuroactive Drugs Prescribed in England and Calculation of the Amount of Each Prescribed Annually

The annual prescription data used in this article were retrieved from the Prescription Cost Analysis (PCA) carried out by the National Health Services (NHS) of the United Kingdom and published by the NHS Business Services Authority (https://www.nhsbsa.nhs.uk/statistical-collections/prescription-cost-analysis-england, accessed on 1 November 2020). The NHS PCAs provide details of the number of items and cost of all prescriptions dispensed in the community, that is, by community pharmacists, appliance contractors, dispensing doctors, and items personally administered by doctors. The present work was based on prescriptions dispensed in England in 2019. These data do not include pharmaceuticals prescribed in hospitals, by private doctors, or purchased via the internet, nor drugs taken or dispensed illegally. Each pharmaceutical included in the PCA is classified within specific chapters of the British National Formulary (BNF). The latter is an annual joint publication of the British Medical Association and the Royal Pharmaceutical Society, and provides up-to-date key information on the selection, prescribing, dispensing, and administration of medicines in the UK. The BNF includes 23 chapters used to classify pharmaceuticals according to their clinical applications. Here we define neuroactive pharmaceuticals as any compound explicitly intended to modulate the central nervous system (CNS), and we propose that CNS-acting drugs have higher probability of perturbing animal behaviour. Hence, to generate a comprehensive assessment of the number and quantity of neuroactive pharmaceuticals beyond antidepressants and anxiolytics, we extracted data for all compounds classified in BNF Chapter 4, “Central Nervous System.” In addition, antihistamines were also included in the analysis due to their well-known ability to modify both fish behaviour [24] and human behaviour [25]. The total amount of active principle prescribed was calculated for each individual preparation as described by [26]. The OpenPrescribing database (https://openprescribing.net, accessed on 1 February 2022) was used to evaluate the regional differences in the prescription of selected classes of neuroactive pharmaceuticals (i.e., antidepressants, anxiolytics, opioid analgesics).

### 2.2. Calculation of Predicted Environmental Concentrations (PECs) in England

Annual prescription data were used to derive predicted environmental concentrations (PECs) (i.e., for surface waters in England, considering a worst-case scenario with 0% removal) as described by the UK Environmental Agency Research and Development Technical Report P390 [27], using the following equation:

Aquatic PEC_Surface Waters_ (g/L) = A × (100 − R)/365 × P × V × D × 100 (1)

where

- A (kg) = predicted amount used per year in England;
- R (%) = removal rate (set to 0 to simulate the worst-case scenario);
- P = number of inhabitants of the country (set to 56,287,000, as indicated by the UK Office for National Statistics-https://www.ons.gov.uk, accessed on 1 December 2020);
- V (m^3^) = volume of wastewater per capita and day (set to 200—default value EMA guideline);
- D = factor for dilution of wastewater by surface water flow (set to 10—default value EMA guideline);
- 100 = conversion factor for percentage.

### 2.3. Prediction of Drug Uptake and Concentration in Fish Plasma 

The PEC values for each compound were used to calculate the concentrations of drugs expected to be present in the plasma of fish exposed to those PECs. Predicted drug plasma concentrations were calculated using the theoretical partition coefficient between water and fish blood based on chemical lipophilicity, as described by Margiotta-Casaluci et al. (2014) [15,28], using the following equations:

Log P_Blood:Water_ = 0.73 × Log K_OW_ − 0.88 (2a)

Log P_Blood:Water_ = 0.73 × Log D_(pH 7_._4)_ − 0.88 (2b)

Fish Steady State Plasma Concentration (F_SS_PC, µg/L) = PEC (µg/L) × P_Blood:Water_ (3)

Log K_OW_ and Log D_7.4_ values for each chemical were retrieved from the ChemSpider database (http://www.chemspider.com, accessed between 1 January 2021 and 1 July 2021) and calculated using the ACD/Labs Percepta Platform-PhysChem Module. 

### 2.4. Estimation of the Pharmacological Risk for Freshwater Fish Species

The pharmacological risk of each compound was estimated by comparing the predicted concentrations of pharmaceuticals in fish plasma (ng/mL) and the human therapeutic plasma concentrations (HTPC), expressed as C_max_ (ng/mL), using the following Equation (4):

Predicted Pharmacological Risk = F_SS_PC/HTPC (4)

The closer F_SS_PC is to HTPC, the higher the risk that the drug may elicit mode-of-action-specific effects in fish comparable to those observed in humans. The risk was classified using the following criteria:

- High risk—F_SS_PC/HTPC ≥ 1
- Medium risk—F_SS_PC/HTPC between 0.1 and 1
- Low risk—F_SS_PC/HTPC < 0.1

These criteria were set using an arbitrary approach informed by pharmacological considerations and were considered as a first-tier interpretation to compare the risk of a high number of compounds. A more refined and advanced risk evaluation using drug-specific considerations was beyond the scope of the present work and was not performed. C_max_ values were retrieved from Schulz et al. (2012) [29] and Berninger et al. (2016) [30], with a few exceptions (as indicated in the Supplementary Data file). 

### 2.5. Evaluation of the Environmental Occurrence of Each Drug 

The environmental occurrence of each pharmaceutical was assessed by examining its presence in PHARMS-UBA, a publicly available database curated by the German Environment Agency (Umweltbundesamt–UBA) (https://www.umweltbundesamt.de/en/database-pharmaceuticals-in-the-environment-0, accessed on 1 February 2022). On the date of access (February 2022), the database contained environmental concentrations of human and veterinary pharmaceutical residues in 61 different types of environmental matrices from 89 countries, extracted from 2062 publications and 240 review articles. The database was also used to extract the measured concentrations of the top 50 most prescribed pharmaceuticals in our list. Specifically, we considered measured concentrations in surface waters reported by publications characterised by “good literature credibility.” The latter is a quality flag (assigned by the database managers to each data entry) that refers to the reliability, plausibility, and applied analytical standards of each publication. Reports associated with poor or unknown credibility were excluded from the analysis. To enhance the source coverage of the analysis, the UBA data were integrated with the assessment of 100 recent papers covering the issue of pharmaceuticals in the aquatic environment, including some with a specific focus on neuroactive pharmaceuticals. These papers were used to evaluate the environmental occurrence (i.e., in rivers) of the 50 most prescribed pharmaceuticals on our list.

## 3. Results

### 3.1. Prescription of Neuroactive Pharmaceuticals in England 

**Figure 1:**
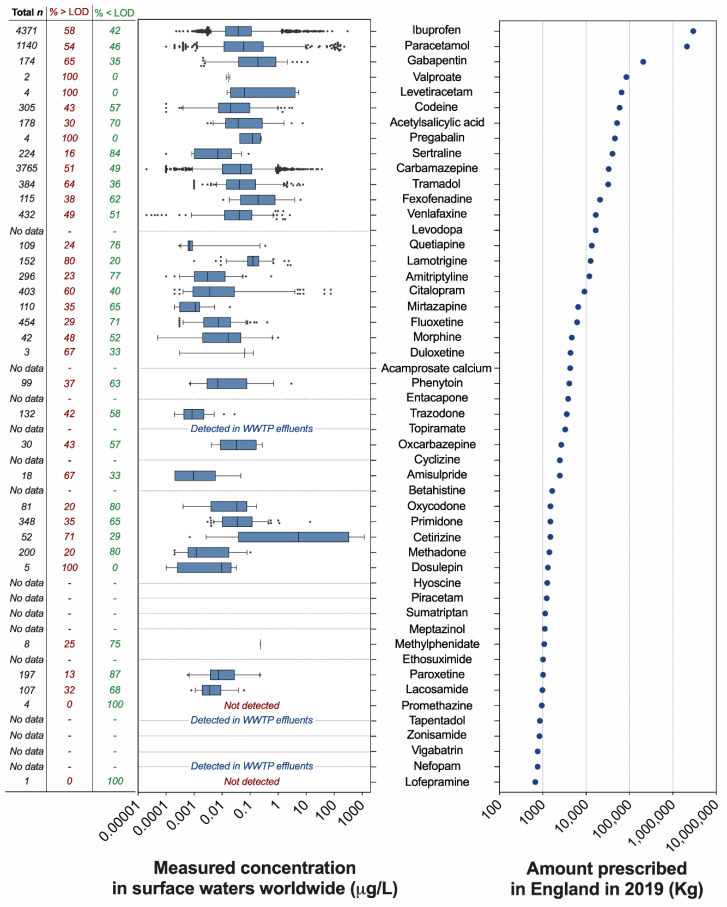
**Top 50 neuroactive pharmaceuticals dispensed in England and their concentrations in surface waters worldwide.** (**Left panel**) Measured concentrations of neuroactive pharmaceuticals in surface waters worldwide (μg/L). The range of concentrations is visualised as box plots, where the limits indicate the 5th and 95th percentiles of the data distribution. Data points outside this range are visualised as individual dots. The vertical line in each box indicates the median value. The data were extracted from the PHARMS-UBA database curated by the German Environment Agency (Umweltbundesamt–UBA) and represent only the values generated by scientific reports classified as “good literature credibility” by the database curators. For each pharmaceutical, the figure indicates the number of available datapoints in the database (first column), the percentage of data above the limit of detection (second column), and the percentage of data below the limit of detection (third column). (**Right panel**) Top 50 neuroactive pharmaceuticals prescribed in England in 2019 and ranked by dispensed amount (kg). The data were generated by analysing the Prescription Cost Analysis (PCA) report (year 2019) provided by the National Health Services (NHS) of England (United Kingdom) and published by the NHS Business Services Authority.

The analysis of the annual prescription data published by the National Health Services (NHS) of England (UK) revealed that 210 different pharmaceuticals acting on the CNS are prescribed to treat a variety of CNS-related conditions, including mental health and sleep disorders, dementia, epilepsy, nausea, and pain. Prescription volumes vary greatly among active pharmaceutical ingredients (Figure 1, Supplementary Data file). Unsurprisingly, the painkillers ibuprofen and paracetamol were the most dispensed compounds in England in 2019, with 2974 and 2122 tonnes, respectively. The third, fourth, and fifth most dispensed compounds were the anticonvulsants gabapentin (208 tonnes), valproate (~85 tonnes), and levetiracetam (~66 tonnes). The most prescribed SSRI antidepressant was sertraline (40.6 tonnes). As a term of comparison, the SSRI fluoxetine (intensively investigated in ecotoxicological studies) was dispensed in much lower volumes (~6.2 tonnes) and was preceded by other antidepressants such as venlafaxine (16.7 tonnes), amitriptyline (11.9 tonnes), and citalopram (9.2 tonnes). Overall, the prescription of 43 neuroactive pharmaceuticals out of 210 exceeded 1 tonne (20%); 50 compounds (24%) were in the range of 100–999 kg, 49 compounds in the range of 10–99 kg (23%), and 36 compounds (17%) in the range of 1–9 kg. Finally, the prescription of 32 compounds (15%) was lower than 1 kg (Supplementary Data file). 

### 3.2. Regional Prescription Trends

**Figure 2:**
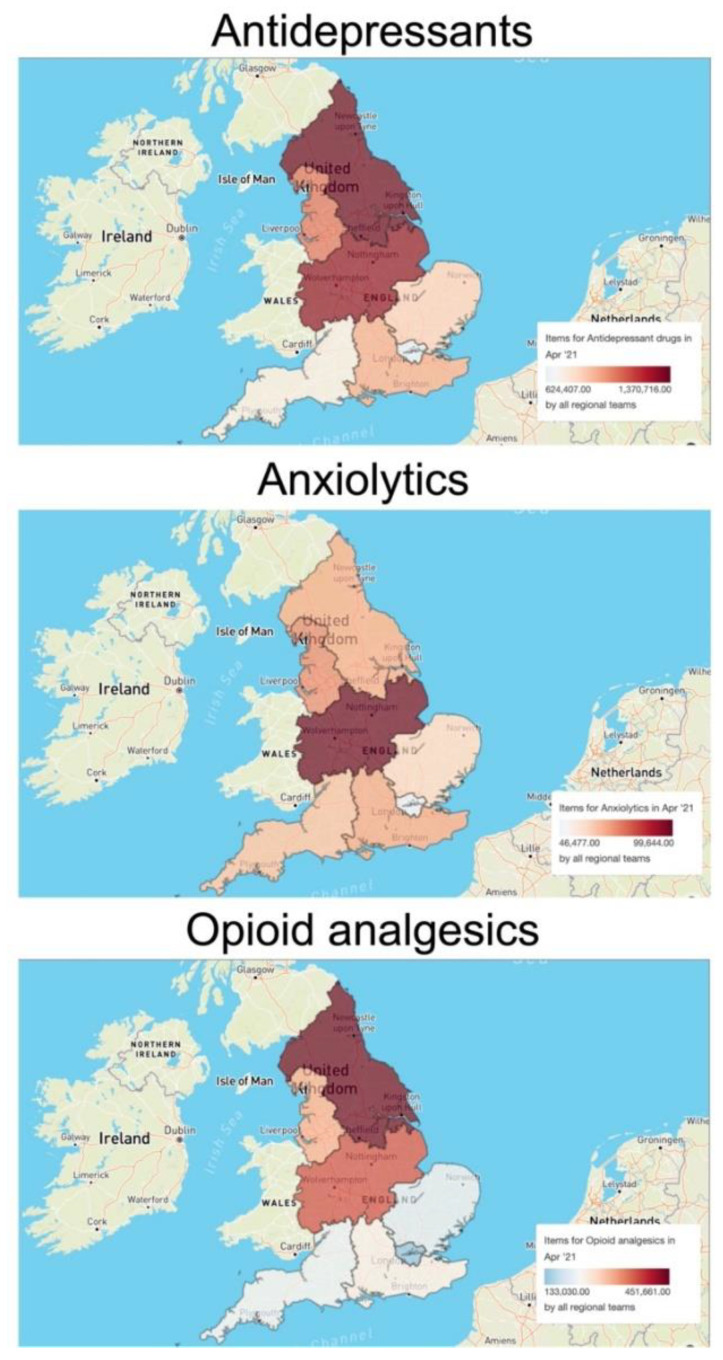
**Regional differences in the prescription volumes of three selected classes of neuroactive pharmaceuticals (antidepressants, anxiolytics, opioid analgesics) in England in April 2021.** The maps and related data were generated using the OpenPrescribing database (https://openprescribing.net/, accessed on 1 February 2022). The volume of each class of pharmaceuticals is expressed as number of items dispensed in April 2021.

The prescription volume of each pharmaceutical plays an important role in determining environmental occurrence and drug concentration in surface waters. To evaluate the significance of regional prescription trends for the interpretation of the environmental risk of pharmaceuticals, we used the OpenPrescribing database to assess the regional differences in prescription volumes in England for three major classes of interest: antidepressants, anxiolytics, and opioid analgesics. As an example, we considered the items dispensed in April 2021. The analysis revealed important region-specific scenarios (Figure 2). For example, the prescription of antidepressants in the North East and Yorkshire Commissioning region (1,370,716 items) and the Midlands Commissioning region (1,297,943 items) appeared to be higher than in the rest of England (e.g., 624,407 items in the London Commissioning region; 744,468 items in the South West Commissioning region). On the other hand, the prescription of anxiolytics was higher in the Midlands region (99,644 items) and lower, but homogenous, in all other areas. Finally, the prescription of opioid analgesics was higher in the Midlands and North England (i.e., a total number of 1,133,000 dispensed items) than in the South England regions (i.e., a total number of 778,050 dispensed items).

### 3.3. Environmental Occurrence of the 50 Most Prescribed Neuroactive Pharmaceuticals

To evaluate the occurrence of the neuroactive compounds identified in our analysis in worldwide surface waters, we extracted relevant data from the PHARMS-UBA database curated by the German Environment Agency, and we integrated this evaluation with the analysis of 100 papers recently published in the field of pharmaceuticals in the environment. A detailed analysis was carried for the 50 most prescribed neuroactive pharmaceuticals (Figure 1), whereas the simple presence or absence in the database was evaluated for the remaining 161 compounds in the list. 

No surface water occurrence data were available in the database for 15 out of the 50 most prescribed neuroactive pharmaceuticals. Three of these 15 compounds were detected in WWTP effluents (topiramate, tapentadol, nefopam). Some of the drugs that have not, as far as we are aware, yet been reported to be present in the aquatic environment are new drugs that have only been in use in the last few years (e.g., nefopam, vigabatrin, zonisamide). Researchers may not have been aware of these drugs when they conducted their analytical studies, and even if they had been, the drugs may not have been present in the water samples they analysed because the drugs were not in use at the time. It is also very likely that some of the drugs in use in the UK in 2019 were not in use in other countries, and hence, water samples collected from rivers in those countries could not have contained those drugs. On the other hand, 33 of the 50 most prescribed neuroactive pharmaceuticals were detected in surface waters worldwide in a wide range of concentrations. In most cases, the reported concentrations were in the ng/L range, and often in the low ng/L range. The median measured surface water concentration exceeded 0.1 µg/L only for cetirizine (5.4 µg/L), fexofenadine (0.19 µg/L), gabapentin (0.19 µg/L), lamotrigine (0.13 µg/L), methylphenidate (0.23 µg/L), and pregabalin (0.12 µg/L). Two compounds, promethazine and lofepramine, were targeted in a small number of samples but were not detected. There were relatively few reports of drugs being present in the µg/L range. However, extremely high concentrations of some of the 50 most prescribed compounds (e.g., carbamazepine, fexofenadine, paracetamol, and tramadol) were reported from rivers in Nigeria [31], where concentrations of carbamazepine and paracetamol were not far below 100 µg/L in some river water samples.

The number of data points available for each pharmaceutical was highly variable and ranged from the 4371 measurements available for ibuprofen to the very few measurements (<5) available for valproate, levetiracetam, pregabalin, duloxetine, promethazine, and lofepramine (Figure 1). In addition to the measured concentrations reported for each compound, we also analysed how frequently each compound was targeted but not detected in the analysed surface water samples. This analysis revealed that the frequency of non-detections was considerable in most cases. Considering the pharmaceuticals associated with 10 or more measurements, the frequency of non-detections ranged from 20% (lamotrigine) to 87% (paroxetine).

Expanding the evaluation of the environmental occurrence to the full list of 210 neuroactive compounds identified in our analysis, 84 were associated with measured surface water concentrations in the PHARMS-UBA database.

### 3.4. Prediction of the Pharmacological Risk for Fish

**Figure 3:**
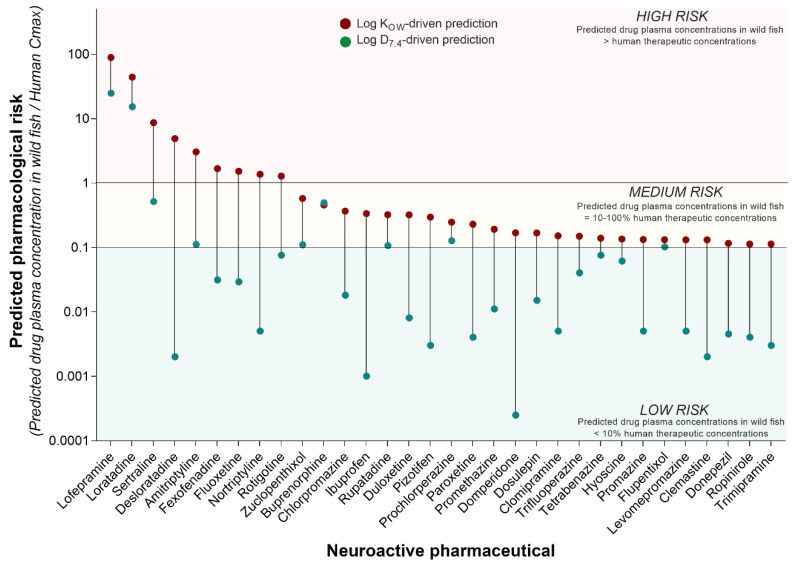
**Predicted pharmacological risk to freshwater fish of neuroactive pharmaceuticals.** The pharmacological risk of the 210 neuroactive pharmaceuticals identified in the present study was estimated by comparing the predicted concentrations of pharmaceuticals in fish plasma (F_SS_PC, ng/mL) and the human therapeutic plasma concentrations (HTPC) expressed as C_max_ (ng/mL). Considering the ratio F_SS_PC/HTPC, values ≥ 1 were classified as “high risk”, values between 1 and 0.1 as “medium risk”, and values < 0.1 as “low risk”. The figure displays all neuroactive pharmaceuticals predicted to have medium and high risk, based on the use of LogK_OW_ for the prediction of drug uptake (red dots). To understand the impact of the use of different partitioning factors on the overall pharmacological risk, the same prediction was also performed using Log D_7_._4_ (green dots).

Although the concentration of pharmaceuticals in surface waters is a key driver of the environmental risk assessment process, it is the concentration of the compound inside the organism (i.e., fish) that determines the pharmacological and toxicological risk. Hence, given two compounds with comparable in vitro pharmacological potency, their comparative in vivo pharmacological risk is determined by their differential tendency to be taken up by the organism, distributed, metabolised, and excreted. To predict the pharmacological risk of each neuroactive compound in our list, here we applied an integrated analysis that involved the following steps. Firstly, we used the annual amount of pharmaceuticals dispensed in England to calculate the related PECs in surface waters. Successively, we used the Fish Plasma Model to predict the drug plasma concentrations resulting from the exposure of fish to those PECs. Finally, the predicted fish plasma concentrations were compared to human C_max_ values to interpret the pharmacological risk posed by each compound. This analysis revealed that nine out of 210 neuroactive pharmaceuticals may reach plasma concentrations in wild fish high enough (i.e., equal to or higher than the human C_max_) to elicit pharmacological effects comparable to those observed in humans in a clinical setting (Figure 3). These drugs were classified as “high risk” and included lofepramine, loratadine, sertraline, desloratadine, amitriptyline, fexofenadine, fluoxetine, nortriptyline, and rotigotine. On the other hand, 23 out of 210 neuroactive compounds were classified as “medium risk”, as they predicted plasma concentrations in wild fish between 10% and 100% of human C_max_ (Figure 3). These predicted sub-therapeutic levels suggest a lower risk of phenotypically observable effects, but they may still be high enough to induce target-mediated effects, especially under conditions of chronic exposure. The classification of the medium-/low-risk threshold was arbitrary and based on expert judgment. More complex drug-specific considerations will be needed to refine the prediction of the pharmacological risk in future studies. 

The predictions of this analysis were generated considering two different partitioning factors for each pharmaceutical, LogK_OW_ and LogD_7_._4_. The risk classification described above was based on the consideration of the use of LogK_OW_ as a key parameter for the prediction of drug uptake in fish. However, the analysis revealed that the predicted pharmacological risk is highly sensitive to the use of different partitioning coefficients, so the predicted risk is lower when the LogD_7_._4_ is used (Figure 3). Considering this scenario, the pharmacological risk of lofepramine and loratadine remained high. The predicted pharmacological risk of the other compounds decreased to a “low risk” classification, with the exception of sertraline, amitriptyline, zuclopenthixol, buprenorphine, rupatadine, prochlorperazine, and flupentixol, which all retained a “medium risk” classification. 

The driving role played by partitioning factors implies that the outcome of modelling exercises based on the Fish Plasma Model should be interpreted with caution, as more sophisticated drug-specific considerations are required for a more rigorous analysis. For example, it is important to note that the two compounds with the highest predicted pharmacological risk are also very hydrophobic (i.e., lofepramine LogK_OW_ = 6.96; loratadine LogK_OW_ = 5.94). Of the 32 compounds with a predicted medium/high pharmacological risk, 12 have a LogK_OW_ between 5 and 6.96, 19 between 3 and 5, and only 1 compound has a LogK_OW_ below 1 (i.e., hyoscine). Prior to the interaction with the biological target (i.e., wild fish), the hydrophobicity of each compound determines its behaviour in the environmental matrix of interest (e.g., in wastewater treatment plants or in rivers), and ultimately its concentration in the different exposure compartments (e.g., water column vs. sediment). Here it possible to observe that the compound with the highest predicted pharmacological risk (i.e., lofepramine) has yet to be detected in surface waters. Hence, despite the predicted pharmacological risk being high, the actual environmental risk in surface waters may still be low. 

### 3.5. Comparison of Predicted versus Measured Concentrations of Pharmaceuticals in UK Surface Waters and Implications for the Prediction of the Pharmacological Risk 

**Figure 4:**
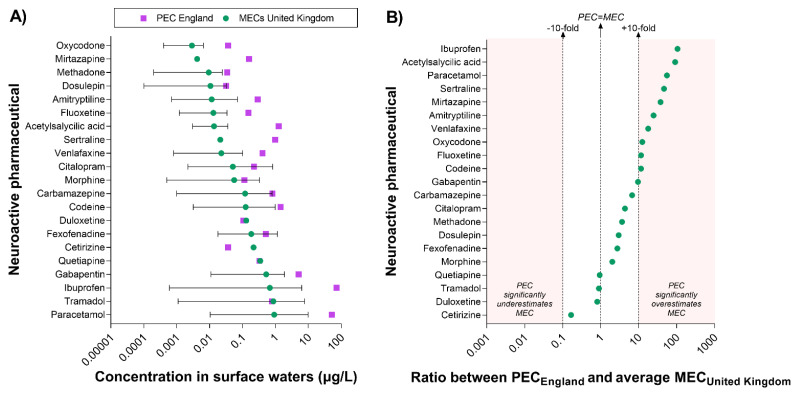
**Comparison between predicted and measured concentrations of neuroactive pharmaceuticals in surface waters in the United Kingdom**. Measured concentrations of neuroactive pharmaceuticals in UK surface waters (MECs)—extracted from the PHARMS-UBA database—were available for 21 compounds. (**A**) This panel displays the range of UK MECs reported for each compound and their average value (i.e., green dots). It is important to note that the latter is not a “true” average MEC, but only the average of the values available in the database, which include single measurements as well as average, minimum, and maximum values. England-specific PECs are indicated by purple squares. (**B**) This panel displays the ratio between PEC_England_ and the average MEC_United Kingdom_ and provides an estimation of the discrepancy between predicted and measured values. To facilitate the interpretation of the data, the vertical dotted lines indicate the level of maximum accuracy (i.e., Ratio PEC/MEC = 1) and the +10-fold and −10-fold range. The red areas indicate when a PEC value overestimates or underestimates the average MEC by more than 10-fold.

The prediction of the pharmacological risk presented in this study is based on the assumption that the predicted concentration of pharmaceuticals in surface waters (i.e., in England; PEC_England_) is representative of the actual concentrations measured in the rivers (i.e., MECs). A significant discrepancy between PEC and MECs would directly affect the accuracy of the predictive model. To evaluate the concordance between the two types of value, we extracted all available UK-specific concentrations measured in surface waters (MEC_United Kingdom_) from the PHARMS-UBA database and compared them with the predicted values (i.e., PEC_England_) (Figure 4). It is important to note that the database does not specify whether the UK values were generated in England or in other regions of the UK. However, we estimated that the majority of those values are likely to refer to water samples collected in England. 

UK-specific data were available for 21 out of the 84 neuroactive pharmaceuticals associated with measured surface water concentrations worldwide. The comparison of PEC and MECs indicated that PECs often overestimate MECs; however, this is not always the case. For example, the PEC of cetirizine was lower than the concentration measured in the environment. Moreover, the PECs of eight compounds (duloxetine, tramadol, quetiapine, fexofenadine, carbamazepine, morphine, citalopram, dosulepin) were within the range of MECs reported in the UK (Figure 4A). 

To better understand the degree of concordance between PECs and MECs, we calculated the ratio between PEC and the average MEC for each compound (Figure 4B). It is important to note that the latter value does not represent a true average of UK MEC, but only the average of the values reported in the PHARMS-UBA database, which include single measurements as well as average, minimum, and maximum values. The analysis revealed a very good concordance for duloxetine, tramadol, and quetiapine. Overall, PECs were within 10-fold the average MEC_United Kingdom_ for 11 out of 21 compounds, whereas they exceeded the 10-fold margin for 10 compounds (i.e., from more to less discrepancy: ibuprofen, acetylsalicylic acid, paracetamol, sertraline, mirtazapine, amitriptyline, venlafaxine, oxycodone, fluoxetine, codeine).

Overall, these results indicate that, despite the overestimation, PEC values for pharmaceuticals can offer a useful first-tier estimation for downstream applications (e.g., the predictive model described in this study), especially when there is a need to compare a large number of compounds. The analytical approach displayed in Figure 4 can be used to refine the estimation of the uncertainty for specific compounds and set ranges of uncertainty tolerability for specific applications.

## 4. Discussion

There is now considerable interest in including behavioural effects in ecotoxicity testing of chemicals [10,32]. If their inclusion is to be of significant use in protecting the aquatic environment from any chemicals that could potentially affect the behaviour of aquatic organisms, the following factors need to be addressed. It is necessary to know which chemicals with the potential to affect behaviour are present in the aquatic environment, and in what concentrations. It is also necessary to know which specific behaviours could be affected by which chemicals, in which organisms, and at which concentrations. Furthermore, ideally the consequences of any behavioural changes would be known. Currently, we are a long way from meeting any of these objectives. Not only is the current relevant literature incomplete, but it is also often contradictory [33]. In this study, we make an initial attempt at identifying the complete repertoire of neuroactive pharmaceuticals likely or already shown to be present in the aquatic environment. We accept that other groups of pollutants (e.g., metals, pesticides) may contain components able to affect behaviour. We also accept that some neuroactive pharmaceuticals may not affect behaviour, and that those that have the potential to do so may affect different behaviours, possibly in different ways. 

### 4.1. Our Findings and Their Implications

The most important result of our study is the finding that a large number (more than 200) of neuroactive pharmaceuticals are in use clinically, and that many of these drugs (*n* = 84) have already been reported to be present in rivers throughout the world. However, this high number is likely an underestimate of the total number of neuroactive substances in use legally and illegally and present in the aquatic environment. This is because our analysis is based only on neuroactive pharmaceuticals prescribed by the National Health Service of the UK, which is just one source of the neuroactive drugs in use. Other sources include over-the-counter painkillers bought from pharmacies or shops without the requirement of a prescription, neuroactive pharmaceuticals prescribed by private medical practitioners, recreational use of neuroactive (illicit) substances, and neuroactive substances formed by metabolism and environmental transformation of parent pharmaceuticals and illicit drugs.

It is very difficult, if not impossible, to estimate the number and amounts of neuroactive substances entering the aquatic environment from these additional sources. It is plausible that the legal additional use, via over-the-counter purchases or private medical practitioners, would add few, if any, pharmaceuticals that are not also prescribed through the NHS. However, the amounts from these additional sources could be substantial, especially for drugs such as ibuprofen, paracetamol, and codeine. In contrast, the situation with illicit recreational drugs is completely different. This is because nearly all illicit drugs are not available in the NHS, and hence they increase the number of neuroactive substances in use and in the environment. These illicit neuroactive substances include cocaine, crack cocaine, MDMA (ecstasy), heroin, various amphetamines, cannabis, various tranquillisers, and ketamine. In addition to those “classic” illicit drugs, new psychoactive substances are constantly appearing [34]. Concentrations of many of these illicit neuroactive substances in the aquatic environment can be in the same range as the concentrations of neuroactive pharmaceuticals taken for medical reasons [35,36,37]. This is readily understood when it is realised that the UK’s National Crime Agency reported that British people consumed 117 tonnes (nearly 120,000 kg) of cocaine in 2019 alone. Others have estimated that 23 kg of cocaine (half a million doses) is taken every day in London, equating to more than 8 tonnes of pure cocaine annually. Whereas much use of illicit drugs is probably spread relatively evenly both spatially and temporally throughout a country such as the UK, special events, such as music festivals, can lead to very irregular “hot spots” of contamination of the aquatic environment [38].

The contribution of neuroactive transformation products, formed either in the patient (metabolites) or wastewater systems and the aquatic environment, is also very difficult to estimate with any confidence, but could be significant. It is undoubtedly the case that at least some of the major neuroactive pharmaceuticals, such as fluoxetine and venlafaxine, and some of the major illicit drugs, such as cocaine, are readily and rapidly transformed (reviewed in Maculewicz et al. (2022)) [39]. Hence, they are present in the aquatic environment [35,36,37,38,39,40,41], often at concentrations similar to, or even exceeding, those of the parent substance. Some of these transformation products definitely possess significant biological activity, although their potencies and specificities are often different to those of the parent substances.

The presence of these neuroactive substances in the aquatic environment would not be of concern if they did not get into aquatic organisms at concentrations high enough to elicit pharmacological effects [15,42,43]. However, most do get into aquatic organisms to some extent, primarily as a consequence of them being hydrophobic [42,44]. A wide variety of human pharmaceuticals have been found in fish [45,46], including a number of neuroactive drugs [21,45], some of which have been found in the blood of wild fish [37,45,47]. A few may even be present in wild fish at concentrations close to, or even at, the human therapeutic concentrations [47]. Our predictive approach based on the integration of pharmacokinetics and pharmacodynamics considerations appears to confirm some of the experimental data, although a more sophisticated and geographically restricted set of predictions would be needed for a more rigorous comparison. For example, Cerveny et al. (2021) [47] identified the antipsychotic flupentixol in the plasma of wild fish (in the Czech Republic) and classified this compound as high risk, as it exceeded human therapeutic concentrations. In our analysis, the same compound was predicted to have medium risk in England. The same authors detected other neuroactive compounds that were predicted to have medium/high pharmacological risk in our analysis, including desloratadine, clomipramine, and pizotifen (in both England and the Czech Republic). Some of the risk classification discrepancies between our analyses and the experimental work of Cerveny et al. (2021) [47] can be explained by the different use of reference C_max_ values. For example, the human C_max_ of desloratadine used in our predictive analysis was 2 ng/mL [29] and led to a medium risk prediction. On the other hand, Cerveny et al. (2021) [47] calculated the pharmacological risk of the same compound using a higher C_max_ of 10 ng/mL, classifying the resulting (experimental) risk as low. Setting the C_max_ value to 2 ng/mL for both studies would have led to a concordant medium risk classification. The list of compounds predicted by our analysis and validated experimentally in the field is further expanded by the work of Malev et al. (2020) [37], who detected four compounds in the blood of wild fish in Croatia that are also associated with high/medium pharmacological risk in the present work (i.e., buprenorphine, loratadine, ibuprofen, sertraline). Overall, this comparison indicates that our predictive approach based on simple drug uptake modelling and human therapeutic considerations confirms it to be a useful strategy for a first-tier risk interpretation and prioritisation exercise. This approach, based on the PECs of parent compounds, may lead to potential overestimations of the risk (Figure 4) [48]. However, the model can easily be refined by incorporating additional parameters, such us human metabolism and excretion, and linked to existing hydrogeological modelling of drug surface water concentrations to achieve a higher spatio-temporal resolution and a more realistic estimation of the risk.

Although, as our results demonstrate, regional differences in neuroactive drug use both within and between countries need to be considered, the basic finding that very many neuroactive substances are present simultaneously in the aquatic environment will be true in all rivers receiving wastewater effluent, as most do. The consequence of that realisation is that, to determine the risk posed by the presence of neuroactive substances, mixture toxicity assessment is required. Appropriate methodology has been developed [49] to enable worthwhile, informative experiments to be designed and their data correctly analysed and interpreted. In addition, Marmon et al. (2021) [50] demonstrated the high potential of using network pharmacology concepts integrated with pharmacokinetics considerations to predict the environmental risk posed by a complex mixture of pharmaceuticals (i.e., 25 NSAIDs). However, formidable obstacles still need to be overcome before it is possible to know whether the presence of complex mixtures of neuroactive substances representative of those present in the aquatic environment pose a significant risk to aquatic organisms. The main current obstacles are identifying the neuroactive substances of greatest concern, the lack of any ecotoxicological data for many of the neuroactive substances known to be present in the aquatic environment, and the limited reproducibility of much of the ecotoxicological data that are available. We discuss each of these three obstacles below.

### 4.2. Current Issues Preventing Significant Progress

At present, it is not possible to know which of the neuroactive substances present in the aquatic environment poses the greatest risk. Although a large number of different neuroactive substances are undoubtedly present, it is quite possible that only a few of them (out of 200+ compounds) contribute the majority of the overall risk posed by the mixture of 210 compounds considered here (see Gustavsson et al. (2017) [51] for an example of this concept based on pesticides). Identifying the toxicity drivers would allow scientists to reduce the complexity of the mixture to an experimentally tractable level and facilitate the regulatory interpretation of the risk. But how do we identify the neuroactive compounds that drive the overall toxicity risk? The predictive integrated approach used in the present study appears to be promising. However, evaluating the accuracy of those predictions would require experimental data. The current ecotoxicological literature is dominated by research on just a few neuroactive pharmaceuticals, including compounds such as fluoxetine and oxazepam, yet as our analysis demonstrates (see Figure 3), some of these may not be the neuroactive substances of greatest concern (e.g., oxazepam). A further complication arises in that the neuroactive substances of most concern in one location may not be those of most concern in another. In this context, mode-of-action-driven grouping of neuroactive compounds may facilitate both mixture toxicity evaluations and read-across approaches, even when experimental data are not available for all the chemicals within the same group.

The majority of the neuroactive substances in use presently, many of which have been shown to be present in the aquatic environment, have not been studied for their fish ecotoxicity. As stated above, a few neuroactive substances have been relatively well studied (e.g., some antidepressants and anxiolytics—see Gould et al. (2021) for a recent review) [52]—although the results of those studies are in some cases inconsistent (see below)—but many are poorly studied or have not been studied at all. This observation may not be surprising, as recent studies have shown that comprehensive environmental toxicity data are lacking for 88% of drugs targeting human proteins [53]. For example, regulatory-relevant fish toxicity data (extracted by Gunnarson et al. (2019)) [53] are available only for five out of the 32 neuroactive compounds predicted to have high–medium pharmacological risk in our predictive analysis (i.e., loratadine, desloratadine, fluoxetine, ibuprofen, duloxetine). This coverage increases (to a limited extent) if we consider non-regulatory relevant academic ecotoxicology studies focused on the characterization of drug-induced behavioural effects in laboratory settings (e.g., sertraline) [22] and biomedical studies. However, the latter are dominated by exposure experiments involving embryo-larvae, and the interpretation of their ecotoxicological relevance remains challenging. On the other hand, chronic exposure studies remain limited. 

The last of the three obstacles that requires discussion is the reproducibility of the available ecotoxicity data, which overlaps with the difficulty to interpret complex behavioural data in a regulatory and decision-making context. It is obvious that it will never be possible to gauge how great the threat that neuroactive substances pose to aquatic organisms is until robust, reliable, repeatable ecotoxicity data are available. Yet the present situation is that there is no agreement on the degree of risk posed by even the most studied neuroactive pharmaceuticals, such as fluoxetine (see Sumpter et al. (2014)) [33]. Some studies report apparent effects when animals are exposed to extremely low, environmentally relevant concentrations of drugs such as fluoxetine; others report effects of low concentrations that are not observed at higher concentrations, e.g., [9,54]; and others report effects only at high concentrations that are well above the environmental range, e.g., [15]. This issue is very well illustrated by the studies published on the possible effects of oxazepam on fish. The same research group has reported that this anxiolytic drug causes behavioural changes in both the laboratory and the field [55] and that it does not [56,57]. We accept that the regulation of behavioural responses is an extremely complex process likely to be modified by many different environmental factors, but nevertheless, if behavioural endpoints are to be utilised in the regulation of chemicals, as some have proposed (e.g., [10]), it is necessary to first substantially improve our understanding of normal behaviour so that any effects of chemicals can be correctly identified. This interpretative challenge is further exemplified by the exercise carried out by Tanoue et al. (2019) [58], where 37 UK and Japan ecotoxicology experts were asked to interpret the significance of a dataset concerning the behavioural effects of tramadol on fish following chronic exposure. Also in that case, the experts reached different conclusions based on the same results. A further interpretative challenge resides in the extrapolation of behavioural effects from the laboratory to the field, as the ecological relevance of typical laboratory-based behavioural testing is currently unclear. 

### 4.3. A Possible Way Forward

What would be an appropriate way to proceed? It is clear that we need to know whether neuroactive substances present in the aquatic environment are adversely affecting aquatic organisms, and if so, which ones. The present *ad hoc* approach based on the behavioural ecotoxicity assessment of one (or very few) neuroactive compound at a time—often selected without an explicit rationale—is too fragmented and seems very unlikely to provide the answer(s) needed. By defining the current eco-pharmacological landscape, our results could be used to inform the design of future research using a pharmacological rationale. Nonetheless, international coordination and cooperation is essential to tackle this scientific challenge in a timely and effective manner. Fostering a wider, international discussion on the best way forward would probably be very beneficial, and it could facilitate the development of coordinated interdisciplinary research initiatives that involve relevant stakeholders in academia as well as industry and regulatory sectors. Positive examples of such ambition are provided by the discussions emerging from recent dedicated workshops and symposia, e.g., Peterson et al. (2017) and Ford et al. (2021) [10,59]. The latter provided, for the first time, a series of consensus statements and useful recommendations aimed at accelerating the regulatory uptake of future behavioural ecotoxicology research. Moreover, a recent review by Bertram et al. (2022) [32] discussed some of the major outstanding questions in behavioural ecotoxicology and proposed a possible way forward. These examples indicate that many scientists around the world are now recognizing the limitation of current practices and are calling for new initiatives aimed at advancing the field in a more organic and coherent manner.

Assessing the ecotoxicity of neuroactive substances using experimental methods remains the biggest challenge. The field of fish behavioural ecotoxicology is currently experiencing an intersection of multiple independent issues (scientific, regulatory, ethical, financial, political) that significantly increases the complexity of the problem. De-structuring such complexity is essential to ensuring progress. The first layer of complexity concerns the ambition to quantify chemical-induced behavioural effects in a reproducible manner. High-throughput multi-dimensional zebrafish behavioural profiling is an established method to identify neuroactive chemicals for drug discovery purposes [60]. This approach is much more complex than the zebrafish behavioural tests commonly used in ecotoxicology research and could be applied to profile the behavioural effects (and the dose response) of hundreds of neuroactive compounds for ecotoxicology applications and generate fish-specific data. However, a limitation of this approach is that it is based on the use of zebrafish embryo-larvae exposed to the test compound for a short period time. We foresee that this approach could be adapted to quantify the behavioural effects of larvae exposed to the drug for longer periods. However, zebrafish larvae acquire a protected status at 120 h post fertilisation; thus, longer exposure times would be associated with much higher ethical costs. More ecologically relevant chronic exposure studies remain scarce, e.g., [15,22,61]. However, even if such studies would be technically feasible, the overall financial and ethical costs would likely be unsustainable or unacceptable. This scenario suggests two possible tractable solutions: (a) to limit chronic exposure studies only to priority compounds (e.g., identified using any prioritization approach, such as the one used here), and (b) to integrate the quantification of behavioural endpoints in current regulatory-relevant chronic toxicity testing, whenever relevant, in order to maximise the amount of information extracted from those in vivo experiments.

The previous points lead us to the second element of complexity, which is the uncertainty surrounding the interpretation of fish behavioural data from a regulatory perspective. To enhance their regulatory relevance, many aspects of laboratory-based in vivo fish behavioural testing require further development and standardisation (e.g., study design, use of positive controls, environmental parameters, ecological relevance of measured endpoints, inter- and intra-laboratory reproducibility, characterisation of baseline behaviour, translation from the laboratory to the field, etc.). On the other hand, behavioural observations of fish in the field can be influenced by numerous confounding factors that hamper the assessment of the causal relationship between drug exposure and effect. If it is necessary, as seems highly likely, to prioritise research in this area, an international discussion on the regulatory and scientific aspects of in vivo behavioural testing (for both adult fish and larvae) should be a high priority; otherwise, research effort will be largely wasted [10,32,56]. 

Laboratory-based fish in vivo testing represents the gold standard to detect chemical-induced behavioural effects, due to the integrated, complex, and dynamic nature of animal behaviour. The considerations provided above are focused on the optimisation and improvement of in vivo fish behavioural testing to enhance its scientific and regulatory value. However, such an in vivo testing strategy would rapidly become incompatible with the recently announced ambition of the US Environmental Protection Agency and European Commission to phase out vertebrate in vivo testing in the next decade or so (i.e., by 2035 in the US) [62]. This political consideration highlights the urgency of supporting research initiatives aimed at understanding the mechanistic basis of chemical-induced behavioural perturbation in fish (and any other relevant vertebrate species). This understanding will be critical to support the identification of a suitable set of new approach methodologies (NAMs) that could be deployed to predict the risk of chemical-induced behavioural alterations without the need to perform animal testing. In this context, the consideration of drug-specific comparative pharmacology, target conservation across species, the in vitro bioactivity profile, and comparative pharmacokinetics (PK) may provide valuable tools to address this challenge [15,50]. In the case of neuroactive pharmaceuticals, this effort can be facilitated by the (generally) advanced understanding of the PK and pharmacodynamic (PD) properties of these compounds in mammals. Based on this understanding, the development and application of multi-dimensional predictive models that integrate both PK and PD (like the one described in this study) can support an effective pharmacology-informed prioritisation and risk assessment of both single compounds and complex mixtures while minimising the reliance of in vivo testing. Thus, the development of predictive in silico/in vitro mechanistic approaches should represent an essential element of any future research strategy in the field of behavioural ecotoxicology.

## Data Availability

The data supporting the results presented in this paper can be found in the Supplementary Data file and on the public databases and repositories indicated in the Section 2.

## Supplementary Materials

The following supporting information can be downloaded at https://www.mdpi.com/article/10.3390/toxics10050233/s1, Supplementary Data File.

## References

[1] Wilson M.W., Ridlon A.D., Gaynor K.M., Gaines S.D., Stier A.C., Halpern B.S. (2020). Ecological impacts of human-induced animal behaviour change. Ecol. Lett..

[2] Doherty T.S., Hays G.C., Driscoll D.A. (2021). Human disturbance causes widespread disruption of animal movement. Nat. Ecol. Evol..

[3] Shannon G., McKenna M.F., Angeloni L.M., Crooks K.R., Fristrup K.M., Brown E., Warner K.A., Nelson M.D., White C., Briggs J. (2016). A synthesis of two decades of research documenting the effects of noise on wildlife. Biol. Rev..

[4] Sprogis K.R., Videsen S., Madsen P.T. (2020). Vessel noise levels drive behavioural responses of humpback whales with implications for whale-watching. eLife.

[5] Kamrowski R.L., Limpus C., Jones R., Anderson S., Hamann M. (2014). Temporal changes in artificial light exposure of marine turtle nesting areas. Glob. Chang. Biol..

[6] Silva E., Marco A., da Graça J., Pérez H., Abella E., Patino-Martinez J., Martins S., Almeida C. (2017). Light pollution affects nesting behavior of loggerhead turtles and predation risk of nests and hatchlings. J. Photochem. Photobiol. B Biol..

[7] Landrigan P.J., Fuller R., Acosta N.J.R., Adeyi O., Arnold R., Basu N., Baldé A.B., Bertollini R., Bose-O’Reilly S., Boufford J.I. (2018). The Lancet Commission on pollution and health. Lancet.

[8] Diamond M.L., de Wit C.A., Molander S., Scheringer M., Backhaus T., Lohmann R., Arvidsson R., Bergman Å., Hauschild M., Holoubek I. (2015). Exploring the planetary boundary for chemical pollution. Environ. Int..

[9] Saaristo M., McLennan A., Johnstone C.P., Clarke B.O., Wong B.B.M. (2017). Impacts of the antidepressant fluoxetine on the anti-predator behaviours of wild guppies (Poecilia reticulata). Aquat. Toxicol..

[10] Ford A.T., Ågerstrand M., Brooks B.W., Allen J., Bertram M.G., Brodin T., Dang Z., Duquesne S., Sahm R., Hoffmann F. (2021). The Role of Behavioral Ecotoxicology in Environmental Protection. Environ. Sci. Technol..

[11] Lucas J.A. (2011). Advances in plant disease and pest management. J. Agric. Sci..

[12] Mars B., Heron J., Kessler D., Davies N.M., Martin R.M., Thomas K.H., Gunnell D. (2016). Influences on antidepressant prescribing trends in the UK: 1995–2011. Soc. Psychiatry Psychiatr. Epidemiol..

[13] Lalji H.M., McGrogan A., Bailey S.J. (2021). An analysis of antidepressant prescribing trends in England 2015–2019. J. Affect. Disord. Rep..

[14] Grabicová K., Grabic R., Fedorova G., Kolářová J., Turek J., Brooks B.W., Randák T. (2020). Psychoactive pharmaceuticals in aquatic systems: A comparative assessment of environmental monitoring approaches for water and fish. Environ. Pollut..

[15] Margiotta-Casaluci L., Owen S.F., Cumming R.I., Polo A., Winter M.J., Panter G.H., Rand-Weaver M., Sumpter J.P. (2014). Quantitative Cross-Species Extrapolation between Humans and Fish: The Case of the Anti-Depressant Fluoxetine. PLoS ONE.

[16] Arnold K.E., Brown A.R., Ankley G.T., Sumpter J.P. (2014). Medicating the environment: Assessing risks of pharmaceuticals to wildlife and ecosystems. Philos. Trans. R. Soc. B Biol. Sci..

[17] Weston J.J., Hugget D.B., Rimoldi J., Foran C.M., Stattery M. Determination of fluoxetine (ProzacTM) and norfluoxetine in the aquatic environment. Proceedings of the Annual Meeting of the Society of Environmental Toxicology and Chemistry.

[18] Brooks B.W., Chambliss C.K., Stanley J.K., Ramirez A., Banks K.E., Johnson R.D., Lewis R.J. (2005). Determination of select antidepressants in fish from an effluent-dominated stream. Environ. Toxicol. Chem..

[19] Brooks B.W., Turner P.K., Stanley J.K., Weston J.J., Glidewell E.A., Foran C.M., Slattery M., La Point T.W., Huggett D.B. (2003). Waterborne and sediment toxicity of fluoxetine to select organisms. Chemosphere.

[20] Brooks B.W., Foran C.M., Richards S.M., Weston J., Turner P.K., Stanley J.K., Solomon K.R., Slattery M., La Point T.W. (2003). Aquatic ecotoxicology of fluoxetine. Toxicol. Lett..

[21] Brooks B.W. (2014). Fish on Prozac (and Zoloft): Ten years later. Aquat. Toxicol..

[22] Valenti T.W., Gould G.G., Berninger J.P., Connors K.A., Keele N.B., Prosser K.N., Brooks B.W. (2012). Human Therapeutic Plasma Levels of the Selective Serotonin Reuptake Inhibitor (SSRI) Sertraline Decrease Serotonin Reuptake Transporter Binding and Shelter-Seeking Behavior in Adult Male Fathead Minnows. Environ. Sci. Technol..

[23] Brodin T., Fick J., Jonsson M., Klaminder J. (2013). Dilute Concentrations of a Psychiatric Drug Alter Behavior of Fish from Natural Populations. Science.

[24] Berninger J.P., Du B., Connors K.A., Eytcheson S.A., Kolkmeier M.A., Prosser K.N., Valenti T.W., Chambliss C.K., Brooks B.W. (2011). Effects of the antihistamine diphenhydramine on selected aquatic organisms. Environ. Toxicol. Chem..

[25] Zannat R., Uddin M.M.N., Rahman M.A., Aklima J., Amin M.M.A. (2016). Antihistamines considerably modulate the cognitive and psychomotor performance of human volunteers. Cogent Psychol..

[26] Runnalls T.J., Margiotta-Casaluci L., Kugathas S., Sumpter J.P. (2010). Pharmaceuticals in the Aquatic Environment: Steroids and Anti-Steroids as High Priorities for Research. Hum. Ecol. Risk Assess. Int. J..

[27] Ayscough N.J., Fawell J., Franklin G., Young W. (2000). Review of Human Pharmaceuticals in the Environment.

[28] Fitzsimmons P.N., Fernandez J.D., Hoffman A.D., Butterworth B.C., Nichols J.W. (2001). Branchial elimination of superhydrophobic organic compounds by rainbow trout (Oncorhynchus mykiss). Aquat. Toxicol..

[29] Schulz M., Iwersen-Bergmann S., Andresen H., Schmoldt A. (2012). Therapeutic and toxic blood concentrations of nearly 1000 drugs and other xenobiotics. Crit. Care.

[30] Berninger J.P., LaLone C.A., Villeneuve D.L., Ankley G.T. (2016). Prioritization of pharmaceuticals for potential environmental hazard through leveraging a large-scale mammalian pharmacological dataset. Environ. Toxicol. Chem..

[31] Ogunbanwo O.M., Kay P., Boxall A.B., Wilkinson J., Sinclair C.J., Shabi R.A., Fasasi A.E., Lewis G.A., Amoda O.A., Brown L.E. (2022). High Concentrations of Pharmaceuticals in a Nigerian River Catchment. Environ. Toxicol. Chem..

[32] Bertram M.G., Martin J.M., McCallum E.S., Alton L.A., Brand J.A., Brooks B.W., Cerveny D., Fick J., Ford A.T., Hellström G. (2022). Frontiers in quantifying wildlife behavioural responses to chemical pollution. Biol. Rev. Camb. Philos. Soc..

[33] Sumpter J.P., Donnachie R.L., Johnson A.C. (2014). The apparently very variable potency of the anti-depressant fluoxetine. Aquat. Toxicol..

[34] Castiglioni S., Salgueiro-González N., Bijlsma L., Celma A., Gracia-Lor E., Beldean-Galea M.S., Mackuľak T., Emke E., Heath E., Kasprzyk-Hordern B. (2021). New psychoactive substances in several European populations assessed by wastewater-based epidemiology. Water Res..

[35] Baker D.R., Kasprzyk-Hordern B. (2013). Spatial and temporal occurrence of pharmaceuticals and illicit drugs in the aqueous environment and during wastewater treatment: New developments. Sci. Total Environ..

[36] Baker D.R., Barron L., Kasprzyk-Hordern B. (2014). Illicit and pharmaceutical drug consumption estimated via wastewater analysis. Part A: Chemical analysis and drug use estimates. Sci. Total Environ..

[37] Malev O., Lovrić M., Stipaničev D., Repec S., Martinović-Weigelt D., Zanella D., Ivanković T., Sindičić Đuretec V., Barišić J., Li M. (2020). Toxicity prediction and effect characterization of 90 pharmaceuticals and illicit drugs measured in plasma of fish from a major European river (Sava, Croatia). Environ. Pollut..

[38] Maasz G., Molnar E., Mayer M., Kuzma M., Takács P., Zrinyi Z., Pirger Z., Kiss T. (2021). Illicit Drugs as a Potential Risk to the Aquatic Environment of a Large Freshwater Lake after a Major Music Festival. Environ. Toxicol. Chem..

[39] Maculewicz J., Kowalska D., Świacka K., Toński M., Stepnowski P., Białk-Bielińska A., Dołżonek J. (2022). Transformation products of pharmaceuticals in the environment: Their fate, (eco)toxicity and bioaccumulation potential. Sci. Total Environ..

[40] Metcalfe C.D., Chu S., Judt C., Li H., Oakes K.D., Servos M.R., Andrews D.M. (2010). Antidepressants and their metabolites in municipal wastewater, and downstream exposure in an urban watershed. Environ. Toxicol. Chem..

[41] Schlüsener M.P., Hardenbicker P., Nilson E., Schulz M., Viergutz C., Ternes T.A. (2015). Occurrence of venlafaxine, other antidepressants and selected metabolites in the Rhine catchment in the face of climate change. Environ. Pollut..

[42] Rand-Weaver M., Margiotta-Casaluci L., Patel A., Panter G.H., Owen S.F., Sumpter J.P. (2013). The Read-Across Hypothesis and Environmental Risk Assessment of Pharmaceuticals. Environ. Sci. Technol..

[43] Huerta B., Margiotta-Casaluci L., Rodríguez-Mozaz S., Scholze M., Winter M.J., Barceló D., Sumpter J.P. (2016). Anti-anxiety drugs and fish behavior: Establishing the link between internal concentrations of oxazepam and behavioral effects. Environ. Toxicol. Chem..

[44] Duarte I.A., Fick J., Cabral H.N., Fonseca V.F. (2022). Bioconcentration of neuroactive pharmaceuticals in fish: Relation to lipophilicity, experimental design and toxicity in the aquatic environment. Sci. Total Environ..

[45] Tanoue R., Nomiyama K., Nakamura H., Kim J., Isobe T., Shinohara R., Kunisue T., Tanabe S. (2015). Uptake and Tissue Distribution of Pharmaceuticals and Personal Care Products in Wild Fish from Treated-Wastewater-Impacted Streams. Environ. Sci. Technol..

[46] Huerta B., Rodriguez-Mozaz S., Lazorchak J., Barcelo D., Batt A., Wathen J., Stahl L. (2018). Presence of pharmaceuticals in fish collected from urban rivers in the U.S. EPA 2008–2009 National Rivers and Streams Assessment. Sci. Total Environ..

[47] Cerveny D., Grabic R., Grabicová K., Randák T., Larsson D.G.J., Johnson A.C., Jürgens M.D., Tysklind M., Lindberg R.H., Fick J. (2021). Neuroactive drugs and other pharmaceuticals found in blood plasma of wild European fish. Environ. Int..

[48] Sumpter J.P., Runnalls T.J., Donnachie R.L., Owen S.F. (2021). A comprehensive aquatic risk assessment of the beta-blocker propranolol, based on the results of over 600 research papers. Sci. Total Environ..

[49] More S.J., Bampidis V., Benford D., Bennekou S.H., Bragard C., Halldorsson T.I., Hernández-Jerez A.F., Koutsoumanis K., Naegeli H., Schlatter J.R. (2019). Guidance on harmonised methodologies for human health, animal health and ecological risk assessment of combined exposure to multiple chemicals. EFSA J..

[50] Marmon P., Owen S.F., Margiotta-Casaluci L. (2021). Pharmacology-informed prediction of the risk posed to fish by mixtures of non-steroidal anti-inflammatory drugs (NSAIDs) in the environment. Environ. Int..

[51] Gustavsson M., Kreuger J., Bundschuh M., Backhaus T. (2017). Pesticide mixtures in the Swedish streams: Environmental risks, contributions of individual compounds and consequences of single-substance oriented risk mitigation. Sci. Total Environ..

[52] Gould S.L., Winter M.J., Norton W.H.J., Tyler C.R. (2021). The potential for adverse effects in fish exposed to antidepressants in the aquatic environment. Environ. Sci. Technol..

[53] Gunnarsson L., Snape J.R., Verbruggen B., Owen S.F., Kristiansson E., Margiotta-Casaluci L., Österlund T., Hutchinson K., Leverett D., Marks B. (2019). Pharmacology beyond the patient—The environmental risks of human drugs. Environ. Int..

[54] Al Shuraiqi A., Al-Habsi A., Barry M.J. (2021). Time-, dose- and transgenerational effects of fluoxetine on the behavioural responses of zebrafish to a conspecific alarm substance. Environ. Pollut..

[55] Klaminder J., Hellström G., Fahlman J., Jonsson M., Fick J., Lagesson A., Bergman E., Brodin T. (2016). Drug-Induced Behavioral Changes: Using Laboratory Observations to Predict Field Observations. Front. Environ. Sci..

[56] Lagesson A., Brodin T., Fahlman J., Fick J., Jonsson M., Persson J., Byström P., Klaminder J. (2018). No evidence of increased growth or mortality in fish exposed to oxazepam in semi-natural ecosystems. Sci. Total Environ..

[57] Fahlman J., Hellström G., Jonsson M., Fick J.B., Rosvall M., Klaminder J. (2021). Impacts of Oxazepam on Perch (Perca fluviatilis) Behavior: Fish Familiarized to Lake Conditions Do Not Show Predicted Anti-anxiety Response. Environ. Sci. Technol..

[58] Tanoue R., Margiotta-Casaluci L., Huerta B., Runnalls T.J., Eguchi A., Nomiyama K., Kunisue T., Tanabe S., Sumpter J.P. (2019). Protecting the environment from psychoactive drugs: Problems for regulators illustrated by the possible effects of tramadol on fish behaviour. Sci. Total Environ..

[59] Peterson E.K., Buchwalter D.B., Kerby J.L., LeFauve M.K., Varian-Ramos C.W., Swaddle J.P. (2017). Integrative behavioral ecotoxicology: Bringing together fields to establish new insight to behavioral ecology, toxicology, and conservation. Curr. Zool..

[60] Kokel D., Bryan J., Laggner C., White R., Cheung C.Y.J., Mateus R., Healey D., Kim S., Werdich A.A., Haggarty S.J. (2010). Rapid behavior-based identification of neuroactive small molecules in the zebrafish. Nat. Chem. Biol..

[61] Thoré E.S.J., Steenaerts L., Philippe C., Grégoir A.F., Brendonck L., Pinceel T. (2019). Improving the reliability and ecological validity of pharmaceutical risk assessment: Turquoise killifish (Nothobranchius furzeri) as a model in behavioral ecotoxicology. Environ. Toxicol. Chem..

[62] Grimm D. (2019). USA EPA to eliminate all mammal testing by 2035. Science.

